# Insulin Resistance Is a Risk Factor for Mild Cognitive Impairment in Elderly Adults with T2DM

**DOI:** 10.1515/biol-2019-0029

**Published:** 2019-07-10

**Authors:** Hongjun Zhao, Chenglong Wu, Xiaoping Zhang, Liping Wang, Jianhong Sun, Fuyuan Zhuge

**Affiliations:** 1 Department of Neurology, The People’s Hospital of Shaoxing City, Shaoxing Zhejiang, No.568 Zhongxingbei Road Shaoxing City Zhejiang Province, 312000 PR China; 2 Department of Gastroenterology, The People’s Hospital of Shaoxing City, Shaoxing Zhejiang, 312000 PR China; 3 Department of Endocrinology, The People’s Hospital of Shaoxing City, Shaoxing Zhejiang, 312000 PR China

**Keywords:** insulin resistance, mild cognitive impairment, elderly adults, T2DM

## Abstract

**Objective:**

The aim of this study was to investigate the clinical effects of insulin resistance (IR) in the development of mild cognitive impairment (MCI) in elderly adults with Type 2 diabetes mellitus (T2DM).

**Methods:**

Seventy-eight patients with T2DM were recruited and divided into MCI group (<26, n=48) and normal group (≥26, n=30) according to the Montreal Cognitive Assessment (MoCA) score. The fasting plasma glucose (FPG), HbA1c, and fasting plasma C-peptide (FPC) were examined and compared between the two groups. The Pancreatic islets function (HOMA-islet) and Insulin Resistance Index (HOMA-IR) were also calculated for the two groups. Using the HOMA-IR and HOMA-islet as the reference, the predicted values for MCI in T2DM patients were calculated by sensitivity, specificity and area under the receiver operating characteristic (ROC) curve.

**Results:**

The MoCA scores were statistically different between the MCI and control groups (23.79±1.15 vs 28.50±1.01, p<0.05). The serum FPG and FPC were 10.38±2.36 mmol/L and 0.79±0.34 ng/mL in the MCI group which were significant different from those of the control group (8.96±2.55 mmol/L and 1.04±0.38 ng/mL; p<0.05). The HOMA-IR and HOMA-islet were 10.08±2.64 and 94.67±29.12 for the MCI group and 8.16±2.46 and 130.30±38.43 for the control group; both were statistically different (p<0.05). The serum HbA1c was 11.02±2.59% and 9.37±2.00% for the MCI and control groups (significantly different with p<0.5). A significant positive correlation was found between MoCA score and HOMA-islet (rpearson=0.44; p<0.001). A significant negative correlation existed between MoCA score and serum HbA1c (r=-0.25; p=0.03). The areas under the ROC curve were 0.70 (0.57~0.82), 0.69 (0.57~0.81), 0.69 (0.57~0.80), 0.72 (0.60~0.84), 0.72 (0.60~0.84) and 0.76 (0.65~0.88) respectively for FPG, FPC, HbA1c, HOMA-IR and HOMA-islet.

**Conclusion:**

Insulin resistance is a risk factor for mild cognitive impairment and can be a biomarker for prediction of MCI in patients with T2DM.

## 1 Introduction

Sporadic Alzheimer’s disease is a common neurodegenerative disease with the main clinical characteristics of memory and cognitive decline, poor spatial orientation, and decline in learning ability [1, 2]. In China, the prevalence of people over the age of 60 is over 5%, and the number of Alzheimer’s disease (AD) patients is over 6 million, which has caused great pressure on society and family [3]. At present, the pathogenesis of sporadic Alzheimer’s disease is not well understood [4, 5]. It is generally believed that the main mechanism is senile plaque, neurofibrillary tangles and lack of specific neurotransmitters in the cortex and hippocampus.

Cognitive dysfunction includes mild cognitive impairment (MCI) and Alzheimer’s disease. MCI is a cognitive function state between normal aging and dementia with the main characters of memory impairment. However, the overall cognitive function is normal without daily life ability impairment. MCI is the second most diagnosed degenerative disease of the nervous system next to cerebrovascular disease. As the aging population in China has increased, the development of MCI and the incidence of AD has also increased rapidly.

At present, a large number of studies have shown that diabetes can cause a wide range of changes in the aspects of neural structures, neurotransmitters, nerve electrophysiology and more [6, 7, 8]. These changes were generally called “diabetic encephalopathy”. At the same time, studies have also demonstrated insulin resistance plays an important role in the development of cognitive impairment and AD. Therefore, we hypothesized that insulin resistance is a risk factor for mild cognitive impairment and can be a biomarker for prediction of MCI in patients with T2DM.

## 2 Material and methods

### 2.1 Patients

Seventy-eight patients with T2DM were recruited from Jan 2015 to May 2017 in the Department of Neurology, The People’s Hospital of Shaoxing City.

**Informed consent**: Informed consent has been obtained from all individuals included in this study

**Ethical approval**: The research related to human use has been complied with all the relevant national regulations, institutional policies and in accordance the tenets of the Helsinki Declaration, and has been approved by ethical committee of The People’s Hospital of Shaoxing City.

**Inclusion criteria** i) All the subjects included in this study were diagnosed with T2DM; ii) The patients’ ages ranged from 55 to 75 years old; iii) Diabetes history was more than 2 years; iv) The patients had normal vision and hearing ability and could receive questionnaires and neurological function tests.

**Exclusion criteria**: i) Patients with congenital mental deficiency; ii) Type 1 diabetes mellitus; iii) Patients with other endocrine system diseases, such as hyperthyroidism, hypothyroidism, systemic lupus erythematosus, etc.; iv) Patients with history of central nervous system injury, such as brain trauma, encephalitis, epilepsy, tumor, infection, severe liver and kidney dysfunction, blood system disease, intellectual disability or drug abuse.

### 2.2 General information of the included cases

The following general information of the included patients was collected: i) gender, ii) age, iii) education level, iv) body weight, v) blood pressure, vi) smoking history, vii) history of cardiovascular and cerebrovascular diseases; viii) course of T2DM, ix) serum levels of fasting plasma glucose (FPG), x) fasting plasma C-peptide (FPC) and xi) Hemoglobin A1c (HbA1c)

### 2.3 HOMA-IR, HOMA-islet calculation

HOMA-IR and HOMA-islet were calculated according to serum FPG and FPC. The equation was: HOMA-IR = 1.5 + FPG × FPC (pmol/L) / 2800; HOMA-islet = 0.27 × FPC (pmol/L) / (FPG-3.5).

### 2.4 MoCA score evaluation

The total Montreal Cognitive Assessment (MoCA) and subset scores of the two groups was acquired according to the MoCA questionnaire [9]. All the patients were tested twice for MoCA and subset scores. The mean score of the MoCA and subset was finally used for the patient’s cognitive impairment evaluation. According to their MoCA score, the included 78 T2DM patients were divided in to the MCI group (<26, n=48) and normal group (≥26, n=30) respectively.

### 2.5 Statistical methods

The data were analyzed by Stata 11.0SE software (http://www.stata.com; Stata Corporation, College Station, TX). The measurement data (MoCA score, HOMA-IR, HOMA-islet, FPG, HbA1c and FPC) were first tested for normal distribution. If the data were normally distributed, it was expressed by mean and standard deviation and compared by Student’s t-tests between the two groups. The enumeration data were expressed by (n, %) and the groups were compared with chi-square tests. The correlation between MoCA score and HOMA-IR, HOMA-islet, and HbA1c were calculated by Pearson correlation test. The significance of detection indexes of HOMA-IR, HOMA-islet, FPG, HbA1c and FPC as biomarkers in the prediction of MCI were evaluated by receiver operating characteristic (ROC) curves, and area under the ROC curve (AUC).

## 3 Results

### 3.1 MoCA of the two groups

**Figure 1:**
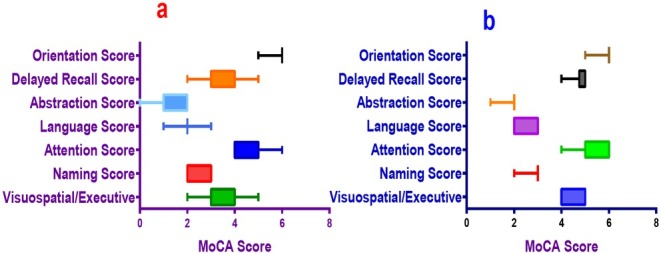
Box plot of MoCA total and subsets scores for the MCI and control groups. a: MoCA total and subset score of the MCI group; b: MoCA total and subset score of the control group

The MoCA scores were 23.79±1.15 and 28.50±1.01 in the MCI and control groups respectively and were significantly different (p<0.05, **Figure 1**). There were significant differences in the subsets of the MoCA for the aspects of Visuospatial/Executive, Naming Score, Attention Score, Language Score Abstraction Score and Delayed Recall Score (p<0.05, **Table 1**).

**Table 1:** MoCA total and subsets scores of the two groups

Items	MCI (n=48)	Control (n=30)	t	P-value
Visuospatial/Executive	3.40±0.79	4.60±0.49	7.46	<0.001
Naming Score	2.69±0.47	2.97±0.18	3.12	0.003
Attention Score	4.75±0.58	5.63±0.56	6.61	<0.001
Language Score	2.00±0.58	2.73±0.45	5.87	<0.001
Abstraction Score	1.50±0.55	1.83±0.38	2.88	0.005
Delayed Recall Score	3.46±0.80	4.77±0.43	8.24	<0.001
Orientation Score	5.98±0.14	5.97±0.18	0.78	0.27
Total MoCA Score	23.79±1.15	28.50±1.01	17.72	<0.001

### 3.2 HOMA-IR, HOMA-islet, FPG, HbA1c and FPC between the two groups

**Figure 2:**
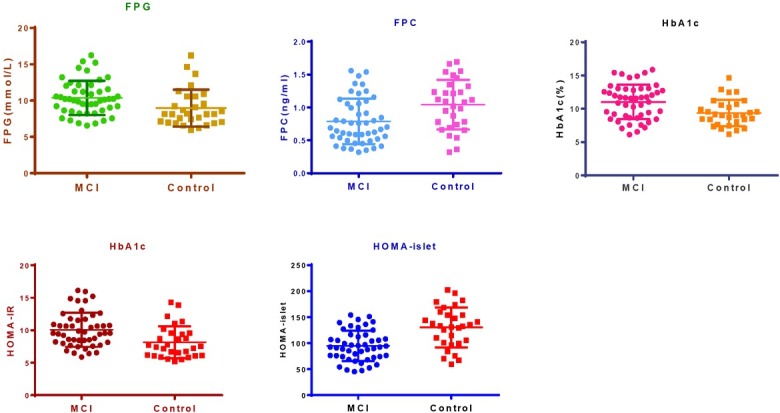
Scatter plot of HOMA-IR, HOMA-islet, FPG, HbA1c and FPC comparison of the two groups

The serum FPG and FPC were 10.38±2.36 mmol/L and 0.79±0.34 ng/mL in the MCI group, which was significantly different from those of control group (8.96±2.55 mmol/L and 1.04±0.38 ng/mL, p<0.05, **Table 2**). The HOMA-IR and HOMA-islet were 10.08±2.64 and 94.67±29.12 for the MCI group and 8.16±2.46 and 130.30±38.43 for the control group and were significantly different between groups (p<0.05). The serum HbA1c was 11.02±2.59% and 9.37±2.00% for the MCI and control groups, with the MCI group significantly higher than the control group (p<0.5). The distribution of HOMA-IR, HOMA-islet, FPG, HbA1c and FPC are shown in **Figure 2**.

**Table 2:** HOMA-IR, HOMA-islet, FPG, HbA1c and FPC comparison of the two groups

Items	MCI (n=48)	Control (n=30)	t	P-value
HOMA-IR	10.08±2.64	8.16±2.46	3.22	0.002
HOMA-islet	94.67±29.12	130.30±38.43	4.64	<0.001
FPG (mmol/L)	10.38±2.36	8.96±2.55	2.50	0.014
HbA1c (%)	11.02±2.59	9.37±2.00	2.97	0.004
FPC (ng/mL)	0.79±0.34	1.04±0.38	30.6	0.003

### 3.3 HOMA-IR, HOMA-islet, HbA1c and MoCA score correlation

**Figure 3:**
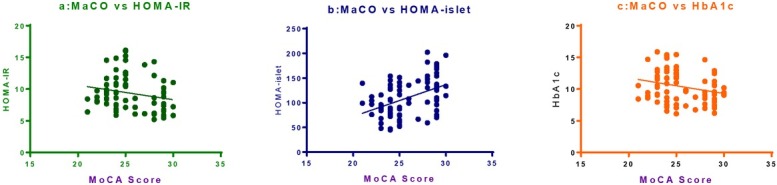
Pearson correlation between HOMA-IR, HOMA-islet, HbA1c and MoCA score

A significant positive correlation was found between MoCA score and HOMA-islet with r_pearson_=0.44 (p<0.001, **Figure 3b**). A significant negative correlation existed between MoCA score and serum HbA1c (r=-0.25, p=0.03, **Figure 3c**). However, there was no significant correlation between MoCA score and HOMA-IR (p>0.05, **Figure 3a**).

HOMA-IR, HOMA-islet, FPG, HbA1c and FPC as biomarkers in predicting MCI

**Figure 4:**
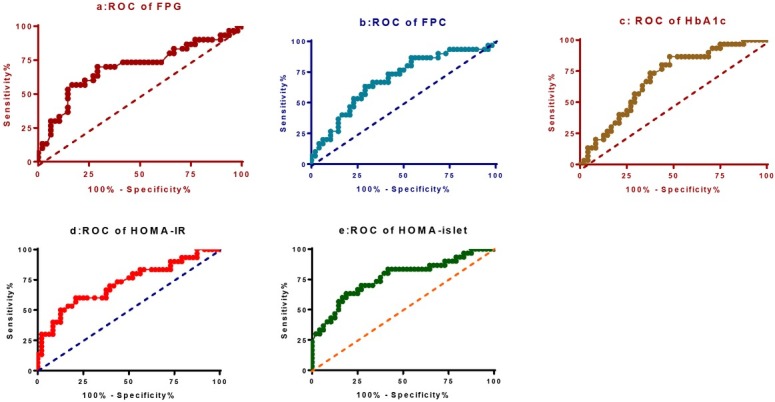
The ROC curves of HOMA-IR, HOMA-islet, FPG, HbA1c and FPC as biomarkers in predicting MCI. a: FPG; b: FPC; c: HbA1c; d: HOMA-IR; e: HOMA-islet

The prediction sensitivities and corresponding 95% CI were 70.00% (50.60～85.27%), 73.33% (54.11～87.72%), 80.01% (61.43～92.29%), 70.00% (50.60～85.27%) and 70.11% (50.60～85.27%) for FPG, FPC, HbA1c, HOMA-IR and HOMA-islet respectively (**Table 3**). The prediction specificities were 70.83% (55.94～83.05%), 58.33% (43.21～72.39%), 56.25% (41.18～70.52%), 60.42% (45.27 ～74.23%) and 72.92% (58.15～84.72%) for the above factors respectively. The AUC were 0.70 (0.57～0.82), 0.69 (0.57～0.81), 0.69 (0.57～0.80), 0.72 (0.60～0.84), 0.72 (0.60～0.84) and 0.76 (0.65～0.88) respectively for FPG, FPC, HbA1c, HOMA-IR and HOMA-islet (**Figure 4**).

**Table 3:** Diagnostic efficacy of HOMA-IR, HOMA-islet, FPG, HbA1c and FPC as biomarkers predicting MCI

Items	Sensitivity % (95%CI)	Specificity % (95%CI) Likelihood ratio	Cut-off	AUC (95%CI)
FPG	70.00 (50.60～85.27)	70.83 (55.94～83.05)2.40	9.22	0.70 (0.57～0.82)
FPC	73.33 (54.11～87.72)	58.33 (43.21～72.39)1.76	0.77	0.69 (0.57～0.81)
HbA1c	80.01 (61.43～92.29)	56.25 (41.18～70.52)1.83	10.78	0.69 (0.57～0.80)
HOMA-IR	70.00 (50.60～85.27)	60.42 (45.27～74.23)1.77	9.01	0.72 (0.60～0.84)
HOMA-islet	70.11 (50.60～85.27)	72.92(58.15～84.72) 2.59	109.2	0.76(0.65～0.88)

## 4 Discussion

Insulin resistance in the nervous system was widely discussed recently. At the beginning of the twentieth century, insulin was thought to be unable to enter into the brain tissue because of the blood-brain barrier. However in the next 50 years, more and more studies demonstrated not only that insulin can enter the brain tissue, but also that the brain tissue was rich in insulin receptors on the surface of neurons. When the number of insulin receptors on the nerve cell membrane was reduced, the dysfunction of insulin appeared and played a role in the metabolism of nerve cells, resulting in the decline of learning, memory and other advanced neurological functions [10].

As for the relationship between hyperglycemia and dementia, a large number of animal and clinical studies have shown that short-term hyperglycemia in experimental animals can cause learning and memory impairment [11, 12, 13]. In recent years, more and more researchers have paid close attention to the relationship between T2DM and MCI. T2DM has been found to be an independent high risk factor for dementia, AD, and MCI [14, 15]. The risk of dementia of T2DM patients is 1.8 times higher than that in non-diabetic people. MCI is the early stage of AD, which can significantly increase the incidence of dementia [16, 17, 18]. Moreover, about 15% of patients with MCI finally develop AD each year.

Tian *et al*. [19] discussed the correlation between insulin resistance (HOMA-IR), Aβ42, plasma IL-1β levels and mild cognitive impairment in T2DM patients. They found that HOMA-IR was negatively correlated with MoCA which was consistent with our results.

At present, there is no effective treatment for AD [20, 21, 22]. In our present study, we included 78 T2DM patients with 48 cases of MCI and 30 normal controls. We found that the serum FPG, FPC, HbA1c, HOMA-IR and HOMA-islet were statistically different between the MCI and control groups, indicating that they may be potential important biomarkers for predicting MCI and indicators for AD. Using these indexes as biomarkers, the prediction value is relatively high for HOMA-IR and HOMA-islet, which could be applicable for clinical practice.

In our present study, we found that HOMA-IR was significantly higher in MCI T2DM patients compared to the control group. This indicates that HOMA-IR is a potential risk factor for the development of MCI. Insulin resistance was characterized by a marked decrease in the sensitivity of the body’s glucose metabolism to insulin, a rise in blood sugar, a large amount of insulin, and a high level of insulin in order to maintain normal levels of blood sugar and then cause hyperinsulinemia. In addition to regulating metabolism and promoting the growth and development of nerve cells, brain insulin plays an important role in advanced intelligence activities such as learning and memory. Sara *et al*. have demonstrated that insulin resistance and hyperinsulinemia were associated with cognitive impairment [23, 24]. Abbatecola *et al*. [25] found that memory can be improved by increasing insulin sensitivity after treatment. These results suggested that the improvement of insulin sensitivity had a protective effect on cognitive dysfunction. However, the mechanism of insulin resistance leading to cognitive dysfunction is not clear, but may be correlated with insulin resistance increasing glycogen synthase kinase-3 activity, and competitive insulin degrading enzymes.

There are still several limitations of our present work: (i) This study was a cross-sectional designed work; patients selection biaswas unavoidable. (ii) The potential confounding factors regarding the biomarkers for prediction of MCI in patients with T2DM were ineluctable which may reduce the strength of the clinical evidence. Therefore, well designed prospective studies are needed for further evaluation of the correlation between insulin resistance and MCI.
